# Broken Windows Theory-Based Spectator Behavior Scale (BWTSBS): a validity and reliability study

**DOI:** 10.3389/fpsyg.2025.1529063

**Published:** 2025-06-05

**Authors:** Sultan Yavuz Eroglu, Erdem Eroglu, Mustafa Can Koc, Laurentiu-Gabriel Talaghir, Teodora Mihaela Iconomescu

**Affiliations:** ^1^Faculty of Sports Sciences, Mus Alparslan University, Mus, Türkiye; ^2^Faculty of Sports Sciences, Istanbul Gelisim University, Istanbul, Türkiye; ^3^Sports Sciences Application and Research Center, Istanbul Gelisim University, Istanbul, Türkiye; ^4^Faculty of Physical Education and Sport, Dunarea de Jos University of Galati, Galati, Romania

**Keywords:** Broken Windows Theory, fans, spectator behavior, validity, reliability

## Abstract

**Purpose:**

The aim of this study is to develop a scale to measure the spectator behaviors of fans and spectators. For this purpose, the research was conducted in two phases.

**Methods:**

The sample of the study consists of 220 fans aged 18 and over who are interested in different sports branches and support various teams across Turkey. Participants were selected from different geographical regions and socio-economic groups to ensure diversity. Stratified sampling method was used in the study. In this method, fans were first stratified according to the sports they support (e.g., soccer, basketball, volleyball) and random samples were selected from each stratum. In this way, it was ensured that each sport branch was represented and the sample better reflected the population.

In the study, Personal Information Form and the Audience Behavior Scale Based on Broken Windows Theory (BWTSBS), which has been validity and reliability study to understand the behaviors of the participants, were used as data collection tools.

**Results:**

In the analysis of the data obtained within the scope of the study, exploratory factor analysis was used for the construct validity of the scale and confirmatory factor analysis was used to verify the structure obtained as a result of this analysis. In this analysis, the extent to which the theoretically determined factor structure overlaps with the data is examined. The goodness of fit indices reveal how well the model fits the data. The CFA used in the study was based on fit criteria such as χ^2^/df, CFI, GFI, AGFI, NFI, IFI, TLI, RMSEA and RMR to evaluate the fit of the model.

## Introduction

In modern capitalist societies, sports fandom makes up a significant portion of individuals' leisure activities. The concept of sports fandom, which has both personal and social roles in fostering a sense of belonging to a group, also plays an important role in promoting social cohesion. However, the spaces where sports fans feel a sociological, psychological, and emotional sense of belonging can sometimes become breeding grounds for violence. For these reasons, individuals may remain in social environments where they experience marginalization and discrimination during their leisure time (Sakar and Sarikan, 2023). Today, sports fandom, which is often practiced as a leisure activity, can sometimes lead to violence as a result of uncontrollable anger. It is well known that violence, driven by the behavior of sports fans, can damage the unity, solidarity, and brotherhood that sports are meant to foster. In this context, spectator violence can be defined psychological, verbal, physical, aggression and violence against players and spectators is a key element of sport, it puts the positive effects on the individual and society on the back burner (Ziyagil et al., 2014).

The Broken Windows Theory was developed by Philip Zimbardo, a psychologist at Stanford University based on an experiment (Ece, 2023). Zimbardo asked people to leave two identical cars without license plates in the Bronx, a poor, high-crime area in New York City, and Palo Alto, a wealthy, low-crime area in California. The car left in the Bronx was looted by vandals within 12 min. Within 24 h, all valuable parts were stripped from the car, and it was further damaged. Afterward, children began playing in the wrecked car. In contrast, the car left in Palo Alto remained untouched for a week. Following this, Zimbardo smashed the windows of the Palo Alto car with a sledgehammer and made further observations. Once the car was partially damaged, it was destroyed by vandals. It was recorded that “respectable white people” were responsible for vandalizing both cars. While vandalism in the Bronx started more quickly due to the nature of community life (where cars are often abandoned, things are stolen, and no one seems to care), Zimbardo's act of breaking the windows in Palo Alto triggered a rapid escalation of vandalism there as well (Welsh et al., 2015). This is not the first study to recognize the potentially harmful effects of social disorder on individuals and society, but it is the first to propose that it may lead to crime. The researchers argue that if even a single instance of disorder emerges, the failure to quickly repair the “broken window” will trigger a chain reaction in the deterioration of the structure. The core idea behind this theory is that unresolved problems in a given environment lead to the perception that formal or informal control mechanisms have broken down. The disorder individuals observe around them directly affects their perception of their social environment. The erosion of norms and values leads to the belief that the physical space they inhabit lacks social control mechanisms. According to the Broken Windows Theory, when people encounter irregularities and negligence, they often fear crime experience fear of crime. Fear of crime has social and individual emotional, cognitive and behavioral dimensions is a multifaceted and complex situation (Nalçacigil, 2020). In this study, it is thought that addressing audience behavior in the context of Broken Windows Theory will have an encouraging effect on future studies and will provide a different perspective to the literature.

## Theoretical justification of broken windows theory in tribune behavior scale development

Tribune environments where sports matches take place are areas where high intensity emotions, group belonging and social interaction are intense. In this context, small-scale norm violations observed in the stands—for example, abusive chants, throwing foreign objects on the pitch, insulting rhetoric toward rival fans—may lead to the legitimization of larger-scale violence or hooliganism over time. This is in line with the chain effect mechanism predicted by the Broken Glass Theory. Therefore, the Broken Windows Theory provides a theoretical basis to explain the impact of environmental cues and observed norm violations on the formation of individual and collective behaviors in the stands. Since the scale focuses on measuring how individuals interpret perceived irregularities in the grandstand and how this affects their own behavior, the Broken Windows Theory is highly contextually and theoretically appropriate.

According to the theory, not intervening in disorder causes individuals to develop the perception that the rules do not work in that environment or that violations go unpunished. In the case of sports tribunes, this situation may cause minor norm violations (e.g., abusive cheering, inappropriate banners, physical contact) to normalize over time and evolve into more serious incidents of tribune violence. Therefore, this scale aims to understand the impact of observed irregularities in the tribune environment on individual behaviors and to explain the mechanism of the formation of tribune behaviors. Broken Windows Theory provides an appropriate and powerful theoretical framework to explain this process and contributes to the systematic evaluation of social norm violations in sports venues.

## Method

### Participants

The aim of the first study was to identify the scope of e-learning and to uncover the scale's structure based on the created item pool. Participants were selected from different geographical regions and socio-economic groups to ensure diversity. Stratified sampling method was used in the study. In this method, fans were first stratified according to the sports they support (e.g., soccer, basketball, volleyball) and random samples were selected from each stratum. In this way, it was ensured that each sport branch was represented and the sample better reflected the population. It is important that the sample size is large enough to ensure that the relationships can be estimated reliably. This number is defined differently according to the reliability of the relationship and the number of significant factors. As a general rule, it is also stated that the sample size should be at least five or even 10 times the number of observed variables (Büyüköztürk, 2002). Accordingly, exploratory factor analysis was performed on 206 participants, and confirmatory factor analysis on 220 participants. Descriptive statistical information about the participants is presented in Table 1.

**Table 1:** Distribution of participants according to descriptive characteristics.

**Groups**	**Frequency (n)**	**Percentage (%)**
**Gender**		
Female	103	50.0
Male	103	50.0
**How fandom is expressed**		
Poor	18	8.7
Average	51	24.8
Good	61	29.6
Excellent	76	36.9
**Following every match of the team**		
Yes	122	59.2
No	84	40.8
**Attendance to matches**		
Yes	61	29.6
No	72	35.0
Sometimes	73	35.4
**Following the team manager's explanations**		
Yes	86	41.7
No	59	28.6
Sometimes	61	29.6
**Following the team Coach's explanations**		
Yes	91	44.2
No	59	28.6
Sometimes	56	27.2
**Football association membership**		
Yes	31	15.0
No	175	85.0

Table 1 shows the distribution of the descriptive characteristics of the participants in the study according to various factors such as gender, expression of fandom, team following, and attendance habits. The gender distribution of the participants was equal, with 50% identifying as male and 50% as female. Regarding how fandom is expressed, 36.9% of the participants described their fandom as “excellent,” while 29.6% categorized it as “good.” The rate of participants following every match of the team stands at 59.2%, whereas 40.8% do not follow all matches. In terms of attendance, 35.4% of participants indicated they “sometimes” attend matches, while 35.0% reported never attending. The percentages of those who follow the statements of the team manager and coach are 41.7% and 44.2%, respectively. Additionally, the majority of participants (85.0%) are not members of a football association. These findings reveal that participants' fan and team-following habits vary, indicating a diverse range of fandom levels.

### Interview and literature review

In the development process of the scale, an online survey was first created for fans in the Eastern Anatolia Region, conducted voluntarily. Following this initial stage, a thorough analysis of the relevant literature was performed. Based on these two applications, the researchers compiled an item pool consisting of 20 items.

### Expert opinion (content-scope validity)

The form developed at this stage was evaluated using a five-point Likert scale, with response options ranging from “strongly disagree” to “strongly agree.” The 20-question trial form was reviewed by three experts in Turkish Language and Literature and three experts in Measurement and Evaluation in Education. Based on the feedback received from these experts, a revised application form consisting of the 20 items was created.

### Data collection tools

The form developed by the researchers includes questions regarding the demographic information of the fans, such as gender, how they express their fandom, whether they follow the team's matches, and their engagement with the statements of coaches and managers.

### Broken Windows Theory-Based Spectator Behavior Scale (BWTSBS)

The initial version of the “Broken Windows Theory-Based Spectator Behavior Scale,” created as part of this research, comprises 20 items. This scale is designed to assess fans' attitudes toward spectator behaviors in the context of Broken Windows Theory and is rated using a 5-point Likert scale.

### Application phase of the scale

Data were collected through an online questionnaire distributed via Google Forms. The form emphasized the scientific nature of the study and encouraged participants to provide sincere and consistent responses to ensure the most accurate results. Additionally, participants were asked to provide an email address for updates regarding the research findings. The data used in the study are not appropriate to be published on an open-access platform due to participant confidentiality and ethical rules. Some demographic and behavioral statements in the data carry the risk of making certain groups identifiable. However, as researchers, the study is planned to share the data with researchers who want to access the data, in accordance with the principle of transparency, if they apply with a statement of commitment in accordance with ethical rules and data protection principles. This was adopted as a solution in line with ethical principles to support the reproducibility of the study.

### Data analysis

The data obtained in the study were analyzed using SPSS 22.0 and AMOS statistical programs. Kurtosis and skewness values were assessed to determine the normality of the scale items' distribution. According to the relevant literature, kurtosis and skewness values between +1.5 and −1.5 (Tabachnick and Fidell, 2013) and between +2.0 and −2.0 (George and Mallery, 2010) are considered indicative of normal distribution. It was determined that the scale items exhibited a normal distribution. For construct validity, both exploratory and confirmatory factor analyses were conducted. Scale reliability was assessed using Cronbach's alpha. Discrimination of the scale was evaluated with independent samples *t*-tests comparing the lower and upper 27% groups. Additionally, independent samples *t*-tests, one-way analysis of variance (ANOVA), and *post hoc* analyses (Tukey and LSD) were employed to examine differences in scale levels based on the descriptive characteristics reported in the study.

## Results

### Reliability analysis

Reliability analysis was performed on 20 items in the scale and Cronbach's Alpha value was 0.857 and 7 items (4, 15, 16, 17, 18, 19, and 20) that negatively affected internal consistency were removed. In the repeated reliability analysis, Cronbach's Alpha value was calculated as 0.937, which shows that the scale has high reliability. When item-total correlations are analyzed in Table 2, item 2 and item 9 have the highest correlations (0.825 and 0.824), and these items contribute significantly to the consistency of the scale. In contrast, item 10 and item 13 have lower correlations and their contributions are relatively weak. There is no major change in Cronbach's Alpha when the items are removed, indicating that the overall structure of the scale is consistent.

**Table 2:** Factor loadings.

**Item**	**Factor loadings**	**Communalities**	**Corrected item-total correlation**	**Cronbach's alpha if item deleted**
item1	0.676	0.457	0.618	0.934
item2	0.866	0.749	0.825	0.928
item3	0.819	0.671	0.769	0.930
item5	0.769	0.591	0.713	0.931
item6	0.786	0.618	0.743	0.930
item7	0.817	0.668	0.771	0.930
item8	0.821	0.675	0.774	0.930
item9	0.866	0.750	0.824	0.928
item10	0.623	0.388	0.567	0.936
item11	0.781	0.610	0.740	0.931
item12	0.690	0.476	0.641	0.935
item13	0.640	0.410	0.592	0.935
item14	0.673	0.453	0.628	0.934

Kaiser-Meyer-Olkin = 0.912; Barlett = p < 0.001; Total Variance = 57.827%; Eigenvalue = 7.517; Cronbach's Alpha = 0.937.

### Exploratory factor analysis

The factor analysis conducted in this study aimed to evaluate the factor structure of the scale, which comprises 13 items. The Kaiser-Meyer-Olkin (KMO) measure was found to be 0.912, indicating that the sample size is adequate for factor analysis. A KMO value above 0.90 suggests that the dataset is highly suitable for this analysis. Additionally, Bartlett's test of sphericity yielded significant results (χ^2^ = 2005.726, *p* < 0.001), demonstrating a substantial correlation among the variables. These findings confirm that factor analysis is an appropriate method for this study.

The communality values indicate the extent to which each item is explained by the factor. Items 9 and 2 exhibit the highest communality values, at 0.750 and 0.749, respectively, suggesting that these items are well represented by the factor. Conversely, item 10 has a lower communality value of 0.388, indicating that this item is less effectively explained by the factor. The first factor accounts for 57.827% of the total variance explained, indicating that the scale can be predominantly represented by a single factor. An analysis of the factor loadings reveals that items 9 and 2 have the highest factor loadings, both at 0.866, demonstrating a strong relationship with the factor. While item 10 has a lower factor loading of 0.623 compared to the other items, it still exhibits a generally acceptable level of factor loading. Varimax rotation was not applied because only one factor was extracted. Rotation is typically employed to enhance the interpretability of the solution when multiple factors are present. In conclusion, the analysis indicates that the scale has a one-factor structure, accounting for 57.827% of the variance in the dataset. These findings demonstrate that the scale is reliable and can effectively be represented by a single factor.

### Confirmatory factor analysis

Confirmatory factor analysis (CFA) is utilized to verify construct validity by assessing how well a model aligns with observed data. This analysis examines the fit of the theoretically determined factor structure to the data collected. Goodness-of-fit indices indicate the extent to which the model fits the data. In this study, CFA was conducted based on fit criteria including χ^2^/df, CFI, GFI, AGFI, NFI, IFI, TLI, RMSEA, and RMR. These criteria confirmed the model's fit, while the factor loadings analysis demonstrated that the majority of the items significantly contributed to the overall structure of the model.

The goodness of fit criteria for confirmatory factor analysis is given in Table 3.

**Table 3:** Confirmatory factor analysis index values.

**Compliance indices**	**Compliance values**	**Limit values**
χ2	227.72	
df	62	
χ2/df (CMIN/DF)	3.67	0 ≤ χ2/df ≤ 5
CFI	0.93	0.80 ≤ CFI ≤ 1.00
GFI	0.90	0.80 ≤ GFI ≤ 0.95
AGFI	0.90	0.80 ≤ AGFI ≤ 0.95
NFI	0.90	0.90 ≤ NFI ≤ 0.95
IFI	0.93	0.80 ≤ IFI ≤ 1.00
TLI	0.91	0.80 ≤ TLI ≤ 1.00
RMSEA	0.08	0.05 ≤ RMSEA ≤ 0.08
RMR	0.06	0.05 ≤ RMR ≤ 0.08

According to the confirmatory factor analysis (CFA) results, the goodness of fit indices of the model are quite satisfactory. The χ^2^/df value is 3.67, which is within the acceptable limits between 0 and 5, indicating that the model is appropriate. The CFI (0.93), GFI (0.90), AGFI (0.90), NFI (0.90), IFI (0.93), and TLI (0.91) values are all within acceptable limits (0.80 ≤ index ≤ 1.00), suggesting that the model has a good fit overall. The RMSEA value is 0.08, and the RMR value is 0.06, which are also within acceptable limits (0.05 ≤ index ≤ 0.08), indicating that the model has an adequate fit.

When examining the Table 4 of factor loadings, it is noted that the critical ratio (C.R.) values for all items are statistically significant (*p* < 0.001). The standardized factor loadings (Std. β) of the items are generally high, with item q8 having the highest factor loading at 0.893, indicating a strong relationship with the factor. The other items also contribute significantly to the overall structure of the model, with factor loadings ranging from 0.600 to 0.839. These findings suggest that the construct validity of the model is strong, and the confirmatory factor analysis confirms the model's fit.

**Table 4:** Factor loadings.

**Articles**			**β**	**Std. β**	**S.E**.	**C.R**.	***P***
q1	<–	F1	1.000	0.657			
q2	<–	F1	1.367	0.839	0.117	11.647	*p* < 0.001
q3	<–	F1	1.285	0.797	0.123	10.420	*p* < 0.001
q4	<–	F1	1.226	0.760	0.122	10.017	*p* < 0.001
q5	<–	F1	1.299	0.759	0.130	10.006	*p* < 0.001
q6	<–	F1	1.274	0.829	0.118	10.756	*p* < 0.001
q7	<–	F1	1.193	0.835	0.110	10.822	*p* < 0.001
q8	<–	F1	1.295	0.893	0.114	11.410	*p* < 0.001
q9	<–	F1	0.812	0.639	0.094	8.631	*p* < 0.001
q10	<–	F1	1.340	0.758	0.134	9.992	*p* < 0.001
q11	<–	F1	1.286	0.667	0.143	8.964	*p* < 0.001
q12	<–	F1	0.963	0.600	0.118	8.155	*p* < 0.001
q13	<–	F1	0.943	0.631	0.111	8.529	*p* < 0.001

#### Distinctiveness

In Table 5, the lower and upper 27% groups were compared to evaluate the discrimination of the scale scores. According to the independent samples *t*-test results, the mean score of participants in the lower 27% group was 13.232, while the mean score of participants in the upper 27% group was 35.464. The difference between these two groups is highly significant (*t* = −18.389, *p* < 0.001). This finding indicates that the scale successfully distinguishes between high and low-performing groups. The results reveal that the discriminative power of the scale is high, and the scores reliably differentiate between different groups.

**Table 5:** Differentiation of scale scores according to lower-upper 27% groups.

**Groups**	**Bottom 27% (*****n*** = **56)**		**Upper 27% (*****n*** = **56)**		**t**	**sd**	***p***
	**Mean**	**Sd**	**Mean**	**Sd**			
Total	13.232	0.426	35.464	9.037	−18.389	110	0.000

Independent samples t-test.

#### Scoring of the scale

The scores assigned to the items in the spectator behavior scale, based on the broken windows theory, are summed to yield a total score. The lowest possible score is 13, while the highest score is 65. A higher score indicates a greater level of spectator behavior, reflecting more positive and engaged behaviors among spectators.

#### Field report

In Table 6, the mean score of the participants' spectator behaviors is reported as 21.869, with a standard deviation of 10.073. The scores range from a minimum of 13 to a maximum of 65, indicating a scale range of 13–65. These findings suggest that there is a notable diversity among participants regarding their spectator behaviors. The results of the analysis conducted to examine the differentiation of the Spectator Behavior scores based on descriptive characteristics are presented below.

**Table 6:** Mean score of spectator behavior.

**Description analyses**	***N***	**Mean**	**Sd**	**Min**	**Max**	**Scale min-Max**
Spectator Behavior Total	206	21.869	10.073	13.000	65.000	13–65

In Table 7, it is observed that spectator behavior scores differ according to demographic characteristics. In terms of gender, the mean score of men (23.602 ± 10.811) is significantly higher than that of women (20.136 ± 9.000) (t = −2.501, *p* = 0.013), indicating that men are more active in spectator behavior. No statistically significant difference was found between the groups for the variable of how fandom is expressed (*p* = 0.137), but it is seen that the average of very good fans is higher than the other groups. In terms of following every game of the team, although the difference is not significant (*p* = 0.094), the scores of those who follow the games are higher. Those who follow the team manager's statements scored significantly higher than those who do not (F = 3.939, *p* = 0.021), with this difference being particularly evident between those who follow the manager's statements and those who sometimes follow them. Similarly, in the case of following the coach's statements, the scores of the followers are significantly higher (F = 3.474, *p* = 0.033). Membership in a football association shows that members have significantly higher spectator behavior scores than non-members (t = 2.408, *p* = 0.049). These findings reveal that various demographic characteristics affect spectator behavior.

**Table 7:** Differences in spectator behavior scores according to descriptive characteristics.

**Demographic Characteristics**	***n***	**Spectator behavior total**
**Gender**		**Mean** ±**SD**
Female	103	20.136 ± 9.000
Male	103	23.602 ± 10.811
t=		−2.501
**p**=		**0.013**
		
**How fandom is expressed**		**Mean** ±**SD**
Poor	18	19.500 ± 12.406
Fair	51	19.686 ± 8.332
Good	61	22.361 ± 7.923
Excellent	76	23.500 ± 11.768
F=		1.866
p=		0.137
		
**Following every match of the team**		**Mean** ±**SD**
Yes	122	22.844 ± 10.347
No	84	20.452 ± 9.546
t=		1.682
p=		0.094
		
**Attendance to matches**		**Mean** ±**SD**
Yes	61	22.721 ± 10.203
No	72	21.528 ± 10.317
Sometimes	73	21.493 ± 9.814
F=		0.308
p=		0.735
		
**Following the team manager's**		**Mean** ±**SD**
**explanations**		
Yes	86	24.105 ± 11.007
No.	59	20.848 ± 10.503
Sometimes	61	19.705 ± 7.448
F=		3.939
p=		0.021
*Post hoc*=		1>3 (p <0.05)
		
**Following the team Coach's**		**Mean** ±**SD**
**explanations**		
Yes	91	23.857 ± 11.006
No	59	20.898 ± 10.731
Sometimes	56	19.661 ± 6.786
F=		3.474
**p**=		**0.033**
*Post Hoc*=		1>3 (p <0.05)
		
**Football association membership**		**Mean** ±**SD**
Yes	31	25.839 ± 12.108
No	175	21.166 ± 9.538
t=		2.408
**p**=		**0.049**
	

F, ANOVA Test; t, Independent Samples T-Test; Post Hoc, Tukey, LSD.

## Discussion

Preventing violence and aggression in the stands is a critical step in promoting the positive development of sports. Despite the implementation of sanctions for negative spectator behaviors, aggressive actions are observed in various forms daily. The literature includes studies exploring the applicability of broken windows theory across different fields, particularly within institutions (Yavuz Eroglu et al., 2022; Kayral, 2019; Temir, 2020). (Polat and Sonmezoglu, 2016) identified environmental and individual factors influencing sports teams' resort to violence. Statements from referees, coaches, and team managers, as well as media portrayals and excessive fan loyalty, can significantly impact spectator behaviors both positively and negatively.

Crucially, inadequate measures against negative spectator behavior contribute to these issues. Therefore, examining fans' behaviors within the framework of broken windows theory using a reliable measurement tool is essential. This research aims to develop a scale for measuring fans' spectator behaviors in this context. The study was conducted in two stages: the first focused on establishing the scale's structure and reliability, while the second validated this structure using a different sample.

As a result of interviews with various team fans and a thorough literature review, an item pool consisting of 20 statements was initially presented to six experts to ensure content validity. Based on the feedback from these experts, the study proceeded with the 20 items. Subsequently, the application phase of the scale began.

In the exploratory factor analysis, the confirmatory factor analysis was performed. The results of the analysis showed that the fit index values (χ^2^/df, RMSEA, CFI, GFI) met the criteria for a good fit as stated in the literature (Steiger, 2000; Thompson, 2004; Kline, 2015).

In the field study, which aimed to examine the differentiation of spectator behavior scores according to descriptive characteristics, it was found that individuals who were defined as good fans, actively followed the matches, and paid attention to the statements of coaches and team managers had higher mean scores. These findings suggest that various demographic characteristics have an impact on audience behavior.

When the literature is examined, there is a scale study called “adaptation of broken windows theory to businesses”, which examines the individual and organizational broken windows theory developed by Bektaş et al. On the other hand, there are studies on the theory of broken windows made in different organizations (Sakar and Sarikan, 2023; Yavuz Eroglu et al., 2022). However, there was no study belonging to the sample group of our study. Therefore, it is thought that the developed scale will make a valuable contribution to the field. Although it was theoretically predicted that grandstand behaviors could be multidimensional, the findings of the exploratory and confirmatory factor analyses showed that a unidimensional structure presented the most appropriate structure for the scale. Factor loadings, explained variance ratio and model fit indices strongly supported this structure. Therefore, in line with the research results, a single-factor structure was accepted instead of a multidimensional structure. This was considered as a simplification process between the theoretical expectation and the analytical findings and explained in the relevant sections of the study. In addition, it was concluded that this unidimensional structure is meaningful and explanatory in terms of showing that tribune behaviors are shaped on the axis of a general perception at a basic level. However, it is thought that possible sub-factors related to the multidimensional structure may emerge in future studies by re-testing the scale on different sample groups. Therefore, although the current findings provide a valid and reliable basis, it is recommended for further research to develop the scale and test the dimensional diversity.

## Conclusion

**Figure 1:**
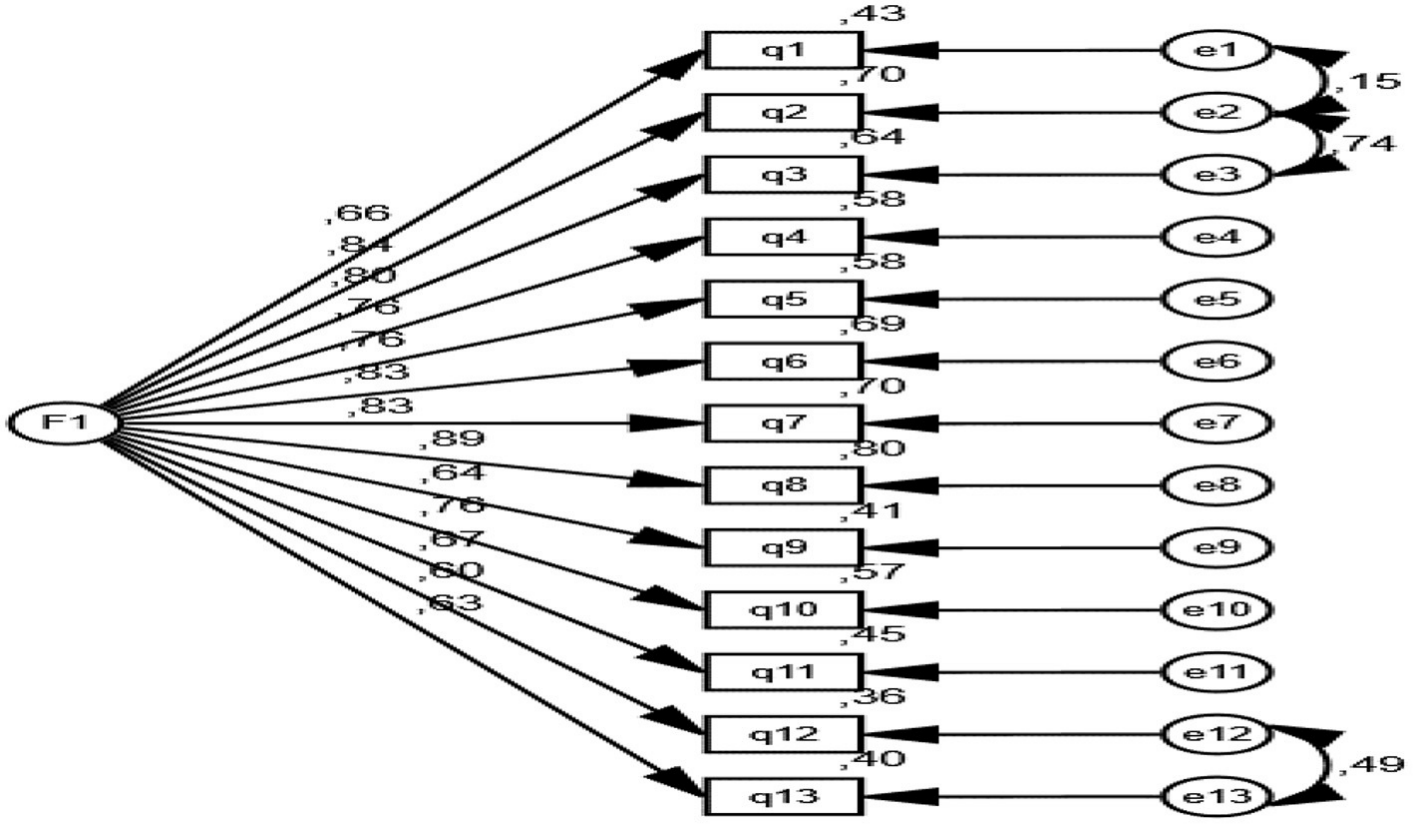
Diagram of confirmatory factor analysis. The goodness of fit criteria for confirmatory factor analysis are given below.

In conclusion, the Broken Windows Theory-Based Spectator Behavior Scale-designed to assess fans' and spectators' behaviors-has demonstrated validity and reliability as a measurement tool, consisting of 13 items and a unidimensional model aligned with the insights derived from the data (Figure 1).

## Data Availability

The data analyzed in this study is subject to the following licenses/restrictions: the data set was not intended to be shared by the authors. If requested, it can be sent by e-mail. Requests to access these datasets should be directed to SY, sultan-yvz@windowslive.com.
